# Working conditions as predictors of retirement intentions and exit from paid employment: a 10-year follow-up of the English Longitudinal Study of Ageing

**DOI:** 10.1007/s10433-015-0357-9

**Published:** 2015-11-27

**Authors:** Ewan Carr, Gareth Hagger-Johnson, Jenny Head, Nicola Shelton, Mai Stafford, Stephen Stansfeld, Paola Zaninotto

**Affiliations:** 1 Department of Epidemiology and Public Health, University College London, London, WC1E 6BT UK; 2 Wolfson Institute of Preventive Medicine, Queen Mary University of London, London, UK

**Keywords:** Job demands, Job resources, Retirement intentions, Work exit, ELSA

## Abstract

Population ageing in Western countries has made delayed retirement and extended working life a policy priority in recent years. Retirement timing has been linked to individual factors such as health and wealth, but less is known about the role of the psychosocial work environment. This paper drew upon longitudinal data on 3462 workers aged 50–69 from five waves of the English Longitudinal Study of Ageing (ELSA). Regression models were used to assess the association of working conditions with preferred timing of retirement and actual work exit. Adjusting for a range of covariates, job demands (aspects of the job requiring sustained physical or psychological effort) were associated with preferences for earlier retirement (by 0.18 years; 95 % C.I. 0.06, 0.31). Decision authority was associated with preferences for later retirement (by 0.38 years; 95 % C.I. 0.23, 0.53) and reduced odds of work exit (OR = 0.93; 95 % C.I. 0.88, 0.97). Low recognition at work was associated with increased odds of work exit (OR = 1.23; 95 % C.I. 1.10, 1.43). There was little evidence of any interactive relationship between demands and resources. Efforts to extend working life should address issues relating to the immediate psychosocial work environment. Providing older workers with increased sense of control, and ensuring contributions are adequately recognised, may delay retirement intentions and the timing of labour market exit.

## Introduction

In recent years, population ageing and improving health at older ages in Western countries have placed political and economic emphasis on the need to reduce early retirement (before statutory pension age) and extend working life (beyond age 50). Employment rates among older workers (ages 50–64) in England are increasing, from 62 % in 2001 to 67 % in 2013 (Redden 2013). Across Europe, however, effective retirement ages (the average age of labour market exit) continue to lag behind statutory ones (i.e. more people stop working before statutory pension age than do after; OECD 2011). With old-age dependency ratios (persons aged 65+ as a proportion of persons aged 20–64) forecast to rise further in coming decades (Eurostat 2015), a better understanding of the antecedents of early labour market exit is imperative.

Retirement decisions have been linked to a range of individual and organisational attributes (Adams and Beehr 2003), but less is known about the role of the psychosocial work environment. Recognising that workplace adjustments represent a modifiable target for policy intervention, this study considers job demands and job resources as potential determinants of extended working. Job demands refer to aspects of the job “that require sustained physical and/or psychological effort” (Bakker and Demerouti 2007), whereas resources are attributes that stimulate personal growth, learning and development, contribute towards the achievement of work goals or reduce job demands (p. 312).

The existing literature on the interplay of demands, resources and subsequent poor health is extensive (Demerouti et al. 2001; Haüsser et al. 2010). However, few studies have considered these factors in relation to retirement, and many of these have focused on specific outcomes (e.g. disability pension) or occupations (e.g. nurses). Of particular relevance, here are the dual psychological processes proposed by Karasek's demand-control model (Karasek et al. 1981), and how these relate to retirement outcomes. In the ‘health impairment’ process, excessive job demands result in high levels of stress, leading in the short term to a state of exhaustion and fatigue (Schreurs et al. 2011), and later, to serious health problems (Landsbergis et al. 1995). The ‘motivational’ process suggests that job resources can motivate employees, resulting in increased levels of work engagement, performance and satisfaction (Bakker 2008).

Working conditions may be related to retirement outcomes via three pathways. High levels of job demands can, by exhausting mental and physical capacity, lead to work overload and subsequent poor health. Given strong evidence showing poor health to predict early retirement and retirement intent (Mortelmans and Vannieuwenhuyze 2013), demands may encourage retirement insofar as they deteriorate health. High demands have also been linked with reduced job satisfaction that can motivate early retirement (Mein et al. 2000) even without the deleterious effects upon health.

A second pathway suggests that job resources may discourage retirement intent by raising levels of work enjoyment and satisfaction. Positive job attributes such as control, social support, career opportunities or financial reward have been shown to be positively associated with job satisfaction (Cheng et al. 2014), work engagement (Xanthopoulou et al. 2009) and subjective well-being (Stansfeld et al. 2013). It has also been shown that employees are less likely to stop working when they enjoy what they are doing or feel fulfilled by their work (Gagné and Deci 2005). Job resources, therefore, may discourage early retirement by enhancing overall job quality.

A third potential pathway arises from the interaction of demands and resources whereby resources influence retirement indirectly by moderating the association between high demand and subsequent poor health. Karasek’s model states that job strain arises from a combination of high job demands and low decision latitude. While empirical support for the interactive demand–resource relationship has been weak (Dollard and Winefield 1998; Landsbergis et al. 1995), job resources may indirectly reduce early retirement intent by weakening the link between job demands and poor health.

## Existing evidence on working conditions and retirement

There has been mixed evidence for the relationship between physically strenuous work and retirement timing. Some studies have found physical demands (e.g. lifting or pushing heavy loads, repeated bending of the neck or back, or standing for prolonged periods) to predict early or health-related retirement (Blekesaune and Solem 2005; Sejbaek et al. 2012), but a recent systematic review (van den Berg et al. 2010) was less supportive, finding a statistically significant association between physical demands and early retirement in just 1/3 studies. With regards to the relationship between psychosocial demands and retirement outcomes, Smeaton et al. (2009) found that older workers in England reporting high levels of work-related stress were more likely to say they plan to retire before state pension age. Laine et al. (2009) used data from the *Finnish Public Sector Study* showing workers reporting high levels of job strain to be 2.60 (95 % C.I. 1.26, 5.34) times more likely to leave work on a disability pension, compared to those reporting low levels of strain (after adjusting for demographic characteristics and health risk behaviour). Other studies, however, have found no support for the association of psychosocial demands upon retirement timing (e.g. Salonen et al. 2003; Zappalà et al. 2008).

Several studies have emphasised job resources (over demands) as the key determinant of retirement outcomes. Hurd and McGarry (1993) found job flexibility and financial rewards (such as pensions or healthcare insurance) to play a greater role in determining extended working (beyond age 62 or 65), compared to physical or mental demands. Retirement intentions have been shown to be influenced by low job control (Sutinen et al. 2005), effort-reward imbalance (Siegrist et al. 2007) and unsupportive workplace norms and supervisors (van Solinge and Henkens 2013). Job control has additionally been linked to labour market exit (Blekesaune and Solem 2005) and disability pension (Vahtera et al. 2010). Other studies have been less supportive, finding no association between job resources and early retirement intent (Sejbaek et al. 2012).

Very few studies have considered the demand–resource interaction in relation to retirement timing. One study found job stress to be a stronger predictor of early retirement when it coincided with low control (Elovainio et al. 2005). Another found high control to reduce the risk of disability retirement due to musculoskeletal disorders (Vahtera et al. 2010).

This paper considers the role of the psychosocial work environment in relation to two outcomes: retirement preferences (preferred number of years until retirement) and labour market exit (moves out of paid employment). Three hypotheses are tested:

### **Hypothesis 1**

Job demands will be associated with preferences for shorter time to retirement and increased probability of labour market exit.

### **Hypothesis 2**

Job resources will be associated with preferences for longer time to retirement and reduced probability of labour market exit.

### **Hypothesis 3**

Job resources will moderate the influence of job demands upon retirement outcomes, such that demands will be less strongly associated with preferences for earlier retirement and work exit when they coincide with high levels of resources.

## Methods

### Data

Data were drawn from five waves of the English Longitudinal Study of Ageing (ELSA), a survey of people aged 50+ living in private households in England (Steptoe et al. 2012). The ELSA sample is drawn from households that previously responded to the Health Survey for England (HSE) in 1998, 1999 or 2001. ELSA respondents were first interviewed in 2002–2003 (*n* = 11,392), with subsequent waves taking place biennially until 2012–2013 (each consisting of a face-to-face interview and self-completion questionnaire). New study members were introduced in 2006/2007 and 2008/2009, recruited from HSE interviews taking place between 2001 and 2006. Ethical approval for ELSA was given by the National Research Ethics Service and all participants gave written consent.

**Fig. 1:**
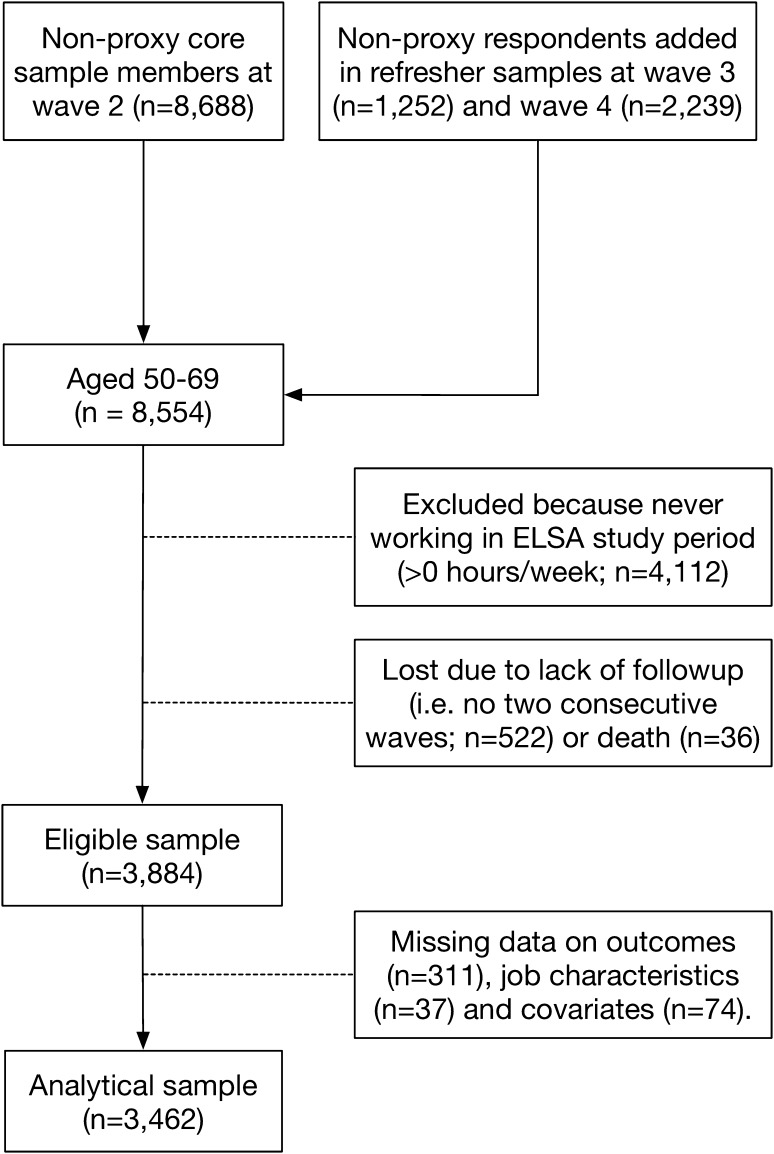
Flow chart of the analytical sample

We omitted the first wave of ELSA (2002/2003), since this included a reduced set of items measuring the psychosocial work environment (compared to later waves), and did not ask respondents about their retirement preferences. We also excluded the most recent wave of ELSA (2012/2013) since respondents’ subsequent work status is unobserved. Our analysis was based, therefore, on 8688 non-proxy respondents who responded at wave 2 (2004/2005) and 3491 respondents who were added as part of the refresher samples in 2006/2007 or 2008/2009. We excluded people outside the age range 50–69 (when joining the study; *n* = 3625), those who were never in paid employment (>0 h/week; *n* = 4112), those lost to death over follow-up (*n* = 36) or with insufficient follow-up data (i.e. individuals who did not respond in at least two consecutive waves; *n* = 522). This produced an eligible sample of 3884 (see Fig. 1).

### Measures

#### Retirement outcomes

Retirement preferences were measured using an item from the self-completion questionnaire that asked respondents “at what age would you like to retire?” From this, we subtracted the respondent’s age at interview to give a measure of preferred years until retirement. Actual exit from employment was defined as a reduction in working hours across two consecutive waves, from >0 to 0 h/week.

#### Job characteristics

Information on working conditions was collected for current employees via a self-completion questionnaire. We derived three scales measuring physical job demands, psychosocial demands and decision authority. For each, we calculated an ordinal alpha reliability score (denoted *α*; Zumbo et al. 2007) based on the polychoric correlation matrix. (1) Physical job demands were measured as the sum of two items. The first asked respondents the extent to which they agreed with the statement “My job is physically demanding” (‘strongly disagree’, ‘disagree’, ‘agree’ or ‘strongly agree’). The second asked about the level of physical exertion in their current job, on a four-point scale from ‘sedentary’ (“You spend most of your time sitting”) to ‘heavy manual’ (“Very vigorous physical activity including handling of very heavy objects”). This gave a continuous score ranging from 1 (low demand) to 7 (high demand; *α* = 0.81).

(2) Psychosocial demands were similarly measured as the sum of two items: working speed (“considering the things I have to do at work, I have to work very fast”) and time pressure (“I am under constant time pressure due to a heavy workload”). Both items were measured on a 4-point scale (‘strongly agree’ to ‘strongly disagree’), giving a score ranging from 1 (low demand) to 7 (high demand; *α* = 0.84). (3) Decision authority was measured as the sum of job control (“I feel I have control over what happens in most situations”) and job autonomy (“I have very little freedom to decide how I do my work”; reversed), giving a score ranging from 1 (low decision authority) to 7 (high decision authority; *α* = 0.77). In addition to the three scales, two binary items were used to measure (4) low social support (“I receive adequate support in difficult situations”) and (5) low recognition (“I receive the recognition I deserve for my work”). For both items, responses of ‘agree’ or ‘strongly agree’ were coded as 0 and ‘disagree’ or ‘strongly disagree’ were coded as 1.

#### Covariates

Age was represented using a spline term with a single knot at age 60. This allowed for the influence of age to be nonlinear, representing the increased probability of work exit around statutory retirement ages (between 60–67 for women and 65–67 for men in our sample). Models were further adjusted for self-rated health (0 = excellent, very good or good; 1 = fair or poor), long-term health problem or disability (that limits the amount or kind of work the respondent can do; 0 = no; 1 = yes) and partner’s employment status (0 = no partner; 1 = partner working; 2 = partner not working; 3 = partner recently retired). This latter category (‘partner recently retired’) identified respondents whose partner was working (>0 h/week) at the previous wave (2 years earlier) but was retired (based on self-reported employment status) at the current wave. This follows past studies (Litwin and Tur-Sinai 2015) showing recent spousal retirement to be predictive of early retirement. Deciles of total income from all sources (employment, benefits, pension, assets and other) were measured at the ‘benefit unit’ level, defined as a single adult or cohabiting couple plus any dependent children (living within the same household).

### Analytical approach

For the continuous measure of retirement preferences (‘preferred years until retirement’) a linear regression model was used, fitted using ordinary least squares estimation in Stata version 13.1 (StataCorp 2013). We considered the association between job characteristics in the current wave (T1) and retirement preferences at the next wave (T2), recognising that these two measures are likely to be endogenous within a single wave of the survey. A robust cluster variance estimator was used to adjust the standard errors to allow for the clustering of observations within individuals.

For labour market exit (a binary indicator of whether the respondent stopped working by the next ELSA wave) we used a discrete-time event history analysis model (Steele et al. 2004). This modelled the conditional probability of work exit in the discrete time periods between successive ELSA interviews (i.e. the hazard rate). The 3462 individuals in the analysis sample generated 7292 person-time observations. These were analysed using a logistic regression model (in Stata version 13.1) with standard errors adjusted with a robust cluster variance estimator. We considered each respondent’s first work exit, ignoring subsequent returns to work (this affected only 41 people).

Sensitivity tests were conducted to test whether the results differed by age or sex and whether findings were sensitive to the chosen cut-point for work exit (0 h/week).

## Results

The analytical sample consisted of 3462 individuals aged 50–69 who were working (>0 h/week) for at least one wave during the ELSA study period. Individuals were omitted due to missing data on retirement preferences (*n* = 311), job characteristics (*n* = 37) and other covariates (*n* = 74). Compared to the excluded sample, the analytical sample was younger (average age of 58.7 vs. 67.4 %; *p* < 0.0001) and contained a smaller proportion of women (52.9 vs. 56.6 %; *p* < 0.0001). The analytical sample was also healthier, with a smaller proportion of individuals reporting poor health (51.2 vs. 67.3 %; *p* < 0.0001) and long-term limiting illness (42.7 vs. 58.9 %; *p* < 0.0001). Descriptive statistics for the analytical sample are given in Table 1.

**Table 1:** Characteristics of the analytical sample

Age [years; mean ± SD (range)]	58.0 ± 4.1 (50.0–69.0)
Female	51.2 %
Poor self-rated health	49.1 %
Long-term limiting illness^a^	41.2 %
Partnership status	
No partner	19.1 %
Partner is working	57.0 %
Partner not working	20.2 %
Partner recently retired	3.6 %
Income decile^b^ [mean ± SD (range)]	7.3 ± 2.4 (1.0–10.0)
Job characteristics	
Physical job demands [scale; mean ± SD (range)]	3.2 ± 1.6 (1.0–7.0)
Psychosocial job demands [scale; mean ± SD (range)]	4.0 ± 1.5 (1.0–7.0)
Decision authority [scale; mean ± SD (range)]	4.8 ± 1.2 (1.0–7.0)
Low social support^c^	25.4 %
Low recognition^c^	30.0 %
Outcomes	
Preferred years until retirement^d^ [mean ± SD (range)]	4.9 ± 6.5 (0.0–70.0)
Work exit next wave^e^	19.4 %
N	3462

^a^Long-term limiting illness or disability that limits amount or kind of work respondent can do

^b^Income measured at ‘benefit unit’ level, defined as a single adult or cohabiting couple plus any dependent children (living within the same household

^c^Percent reporting ‘disagree’ or ‘strongly disagree’

^d^Retirement preferences measured at the next ELSA wave

^e^Percent not working (0 h/week) at the next wave, given employment (>0 h/week) at the current wave

Minimally (age, sex) and additionally adjusted (age, sex, income, self-rated health, limiting long-term illness, partner’s employment status) estimates for the influence of job characteristics upon retirement preferences are presented in Table 2. These were estimated for (a) each job characteristic separately and (b) all job characteristics simultaneously. Considered separately, three out of five job characteristics were significantly associated with retirement preferences at the next wave, after full adjustment. Psychosocial job demands were, per unit increase in the summed score (range 1–7), associated with preferences for retirement 0.25 years earlier (95 % C.I. −0.37, −0.13). Decision authority was, per unit increase, associated with preferences for retirement 0.41 years later (95 % C.I. 0.28, 0.55). Low recognition at work (‘disagree’ or ‘strongly disagree’ compared to ‘agree’ or ‘strongly agree’) was associated with preferences for retirement 0.40 (95 % C.I. 0.07, 0.73) years earlier. When all job characteristics were tested simultaneously, only psychosocial demands (*β* = −0.18; 95 % C.I. −0.31, −0.06) and decision authority (*β* = 0.38; 95 % C.I. 0.23, 0.53) remained statistically significant predictors of retirement preferences.

**Table 2:** Coefficients for retirement preferences (preferred years until retirement) measured at the next ELSA wave

	Minimally adjusted^a^						Additionally adjusted^b^					
	*β*	*β*	*β*	*β*	*β*	*β*	*β*	*β*	*β*	*β*	*β*	*β*
Physical demands	−0.04					0.01	−0.04					0.01
	(−0.15, 0.08)					(−0.11, 0.12)	(−0.15, 0.08)					(−0.11, 0.13)
Psychosocial demands		−0.26***				−0.19**		−0.25***				−0.18**
		(−0.38, −0.14)				(−0.31, −0.06)		(−0.37, −0.13)				(−0.31, −0.06)
Decision authority			0.43***			0.39***			0.41***			0.38***
			(0.29, 0.56)			(0.24, 0.54)			(0.28, 0.55)			(0.23, 0.53)
Low social support				−0.28		0.31				−0.23		0.32
				(−0.64, 0.08)		(−0.12, 0.74)				(−0.59, 0.12)		(−0.10, 0.75)
Low recognition					−0.45**	−0.07					−0.40*	−0.05
					(−0.78, −0.12)	(−0.47, 0.32)					(−0.73, −0.07)	(−0.45, 0.35)
Individuals^c^	3462	3462	3462	3462	3462	3462	3462	3462	3462	3462	3462	3462

Robust cluster 95 % confidence intervals in parentheses

^a^Adjusted for age and sex

^b^Adjusted for age, sex, income decile (at the benefit unit level), poor self-rated health, limiting long-term illness, and partner’s employment status

^c^Person-wave observations = 7822

*** *p* < 0.001; ** *p* < 0.01; * *p* < 0.05

Considered separately, three out of five job characteristics were significantly associated with work exit, after full adjustment (Table 3). Decision authority was, per unit increase in the summed score, associated with reduced odds of work exit (OR = 0.91; 95 % C.I. 0.86, 0.95). In practical terms, workers who reported high decision authority (a score of 7) were 8.6 % less likely to stop working, compared to those reporting low decision authority (a score of 1). Conversely, increased odds of work exit were found for low social support (OR = 1.25; 95 % C.I. 1.09, 1.44) and low recognition (OR = 1.34; 95 % C.I. 1.17, 1.53). Workers who ‘disagreed’ or ‘strongly disagreed’ with the statements on social support or recognition were 3.2 and 4.1 % more likely, respectively, to stop working between two consecutive ELSA waves. When testing all job characteristics simultaneously, only decision authority (OR = 0.93; 95 % C.I. 0.88, 0.97) and low recognition (OR = 1.23; 95 % C.I. 1.10, 1.43) remained statistically significant.

**Table 3:** Odds ratios for work exit next by next ELSA wave

	Minimally adjusted^a^						Additionally adjusted^b^					
	OR	OR	OR	OR	OR	OR	OR	OR	OR	OR	OR	OR
Physical demands	0.99					0.98	0.99					0.98
	(0.96, 1.03)					(0.94, 1.02)	(0.95, 1.02)					(0.94, 1.01)
Psychosocial demands		1.02				0.98		1.02				0.99
		(0.97, 1.06)				(0.94, 1.03)		(0.98, 1.06)				(0.95, 1.04)
Decision authority			0.90***			0.92**			0.91***			0.93*
			(0.85, 0.94)			(0.87, 0.96)			(0.86, 0.95)			(0.88, 0.97)
Low social support				1.29***		1.10				1.25**		1.08
				(1.12, 1.47)		(0.94, 1.28)				(1.09, 1.44)		(0.92, 1.27)
Low recognition					1.36***	1.23**					1.34***	1.23**
					(1.20, 1.56)	(1.10, 1.43)					(1.17, 1.53)	(1.10, 1.43)
Individuals^c^	3462	3462	3462	3462	3462	3462	3462	3462	3462	3462	3462	3462

Robust cluster 95 % confidence intervals in parentheses

^a^Adjusted for age and sex

^b^Adjusted for age, sex, income decile (at the benefit unit level), poor self-rated health, limiting long-term illness and partner’s employment status

^c^Person-wave observations = 7,292

*** *p* < 0.001; ** *p* < 0.01; * *p* < 0.05

All two-way interactions between job demands and job resources were tested, adjusting for other job characteristics and individual covariates. With one exception, no statistically significant interaction effects were observed (at the 5 % level; see Table 4). Among workers reporting low levels of psychosocial demand, those receiving low levels of social support were more likely to stop working, compared to workers reporting higher levels of support (predicted probabilities of work exit for low and high support = 24.3 and 18.3 %, respectively). However, this was only borderline significant (*p* = 0.045), and no corresponding effect was observed for workers reporting high levels of psychosocial demand (predicted probabilities of work exit for low and high support = 19.3 and 18.6 %, respectively).

**Table 4:** Adjusted Wald test statistics for demand–resource interactions

		Retirement preferences		Work exit	
		*F*	Two-sided *p* value	*χ* ^2^	Two-sided *p* value
Physical job demands	× Decision authority	3.14	0.077	0.41	0.520
	× Low social support	0.02	0.895	0.97	0.325
	× Low recognition	0.08	0.773	0.59	0.443
Psychosocial demands	× Decision authority	0.68	0.410	0.79	0.375
	× Low social support	0.30	0.583	4.02	0.045
	× Low recognition	1.75	0.186	0.77	0.380

Wald test statistics adjusted for clustering of repeated observations within individuals

Sensitivity tests were conducted to test whether the influence of job characteristics differed by age or sex. Physical and psychosocial job demands had a stronger downward influence upon retirement preferences as age increased (*χ* ^2^ = 10.22 and 15.73, respectively; *p* < 0.01 on 2 df), but no other differences by age were found. No differences were found by sex, for either outcome. We further tested whether the chosen cut-point for work exit (0 h/week) influenced our findings. The direction and substantive interpretation of results did not change whether this cut-point was set at 0, 5, 10, 15, or 20 h/week.

## Discussion

In this analysis of a nationally representative sample of 3462 older workers in England, we found no evidence of an association between job demands (either physical or psychosocial) and the probability of work exit, but psychosocial demands were predictive of preferences for shorter time until retirement. In contrast, good evidence was found to support the hypothesis that job resources predicted preferences for longer time until retirement and reduced probability of work exit. When adjusting for age and sex, all three measures of job resource (decision authority, low social support and low recognition) were associated with the probability of work exit, as hypothesised, while decision authority and low recognition were predictive of retirement preferences. In the additionally adjusted models, only decision authority and low recognition remained statistically significant. All job characteristics had a stronger influence when considered separately which, given high correlations between the different measures, was to be expected. Workers reporting high levels of decision authority are likely to also enjoy high levels of social support and recognition. We found very little support for our third hypothesis, the interactive relationship between demands and resources. Low psychosocial demands were more strongly associated with work exit if workers reported low social support (compared to high support), but this was only borderline significant (*p* = 0.045) and no corresponding effect was observed for high psychosocial demands.

We found that decision authority and low recognition predicted retirement preferences as well as work exit. However, although psychosocial demands were predictive of wanting to retire sooner, they had no influence upon actual exit probabilities. This is consistent with previous studies showing discrepancies between retirement intentions and behaviours (Solem et al. 2014; Dal Bianco et al. 2015). Workers may be forced to retire earlier than they would like because of poor health or caring responsibilities. Conversely, working life may extend beyond preferred retirement age due to financial insecurity or lack of pension eligibility. That retirement decisions are constrained by individual circumstances is particularly relevant when considering the role of the work environment. While adjusting for income and health, our model of the probability of work exit assumed that individuals had an equal capacity to retire. Our results may underestimate the influence of the work environment, therefore, since only a subset of workers experiencing high demands or low resources will be able to act upon their preferences.

Our findings are consistent with past studies showing no association between job demands and retirement timing (Salonen et al. 2003; Zappalà et al. 2008). The results for decision authority (Blekesaune and Solem 2005) and work recognition (Thorsen et al. 2012) also support those from previous studies. A key contribution of this study was to test the demand–resource interaction in relation to retirement timing. Here, our findings are at odds with past studies on retirement. Elovainio et al. (2005) found support for a demand–control interaction, but their sample incorporated a wider age range than our study (20–65 rather than 50–69) and consisted of Finnish healthcare employees only, rather than the nationally representative sample employed here. Instead, our findings are more consistent with the broader demand–resources literature (e.g. Dollard and Winefield 1998), which tends to support the additive but not interactive effects of demands and resources.

This study is one of the first to consider working conditions and retirement outcomes among a large, longitudinal and nationally representative sample of older workers in England. With the exception of some Scandinavian studies (e.g. Vahtera et al. 2010), past research has often relied upon small sample sizes or focused within particular institutional settings. Other strengths are that job characteristics were measured repeatedly and it was possible to adjust for several potential covariates.

In terms of limitations, our analysis relies on a few simple measures of the work environment. Such measures have been shown to have acceptable validity (Leineweber et al. 2010), but the multi-item scales employed in past studies would provide better coverage of the constructs of interest. The analysis was also limited to considering each respondent’s first observed transition out of work, precluding later returns to work. Job characteristics were measured at older ages only (50+) despite past research showing retirement timing to depend upon occupational exposures across the life course (Liebermann et al. 2013). Sample attrition represents another important limitation. The analytical sample was younger and healthier than excluded respondents. ELSA respondents who are still working at ages 50+ are likely to enjoy more favourable working conditions and be better educated compared to those who exited the labour market before age 50. Our results are generalizable only to workers aged 50–69 living in England and Wales.

These results are important within the context of the UK and European policies to promote extended working. They suggest that workplace modifications to improve the psychosocial work environment can delay retirement timing by moderate but statistically significant amounts. In our results, increases in decision authority (from low to high) were associated with preferences for retirement 2 years later. This is comparable to rises in compulsory retirement age proposed in Europe (Sinclair et al. 2013) and the US (General Accounting Office 2011).

At the same time, we would not wish to overstate the potential for change. Given heterogeneous working arrangements and relations, and the fluctuating influence of macro-economic circumstances, such improvements may be difficult to achieve. Moreover, as noted above, retirement timing is constrained by factors such as poor health or financial need that may curtail or extend working life irrespective of the work environment. Decision authority and work recognition therefore represent important targets for policy, but only insofar as these factors can be successfully modified. Recent large-scale interventions (Hasson et al. 2012; Gilbert-Ouimet et al. 2015) suggest that improvements to the psychosocial work environment are feasible, but further research is needed to develop and test such interventions.
